# The Case for Neonatal Specialist Transport Teams

**DOI:** 10.1016/j.jpedcp.2024.200105

**Published:** 2024-03-16

**Authors:** Lee Collier, Burak Salgin

**Affiliations:** 1 Queen Mary University of London, William Harvey Research Institute, Faculty of Medicine & Dentistry, John Vane Science Centre, Charterhouse Square, London, United Kingdom; 2 Neonatal Intensive Care Unit & London Neonatal Transfer Service (NTS), The Royal London Hospital, Barts Health NHS Trust, London, United Kingdom; 3 Queen Mary University of London, Blizard Institute, Barts and The London School of Medicine and Dentistry, London, United Kingdom

Interfacility transport of neonatal patients is a regular occurrence, with approximately 68 000 transfers per year in the US^1^ and 15 000 in the United Kingdom.^2^ Efforts to improve the transport of neonatal patients began more than a century ago^3^ and has evolved toward provision of specialist ground and air ambulances, incubators and associated equipment, and training of personnel. Research into vehicles and equipment has been carried out using a variety of models^4^; however, producing good evidence for staffing of neonatal transport teams is problematic. Although some early studies are frequently quoted as evidence for the benefit of neonatal specialist (NS) teams,^5^ a recent Cochrane systematic review of evidence for morbidity and mortality reduction found no studies that met their inclusion criteria.^6^ Evidence for the superiority of NS teams may affect case-by-case decision-making, but perhaps more importantly would inform longer-term budgetary and commissioning decisions for regional and national service planning. Therefore, any addition to the scanty evidence base is welcome.

There are broadly 3 categories of teams that may carry out a neonatal transfer: an established regional NS team; an ad-hoc nonspecialist team consisting of pediatric staff from the referring hospital; and a regional non-neonatal team such as a prehospital or HEMS (Helicopter Emergency Medical Service) team. There are a variety of models for a NS transport team, but in general such teams should include a distinct identity and governance structure whether the team is standalone, hosted within a neonatal hospital unit, or part of a larger transport/retrieval service; nurses, physicians, and allied health professionals with significant neonatal intensive care expertise and specific training for transport practice; attending (consultant) neonatal physician supervision; standardized equipment with team expertise to operate; and ideally dedicated road and air vehicles.

The skillset and roles of health care professionals differ by country, with respiratory therapists taking a key role in airway and ventilation management in North America but not in most of the rest of the world. Similarly, the role of paramedics is inconsistent across health care systems globally. NS teams therefore often consist of 2 or 3 neonatal staff with different levels of seniority, including physicians, nurses, respiratory therapists, and paramedics. There may be a fixed team composition within a particular service or consideration of skill mix on a case-by-case basis.

For parents, transport of their newborn baby is uniquely stressful, raising concerns around separation from their baby and lack of information or communication.^7^ The ideal neonatal transport team would therefore not only be highly skilled and experienced in the clinical aspects of a transfer but have the cognitive space and practical capacity to support accompanying parents and implement family-centered innovations such as kangaroo care during transport.^8^

In this study, Gardiner et al present their findings from a unique opportunity to compare the performance of NS teams with ad-hoc non-neonatal specialist (NNS) teams.^9^ The decision to use the NS or NNS team was based on the availability of an aircraft close to the referring center, in which case the NNS team was used, or the availability of a faster jet close to the specialist center, in which case the NS team was used. Although not truly random, aircraft availability is decoupled from clinical decision-making, and the authors make it clear that aeromedical logistics was always the deciding factor. There were 119 infant transports during the study period, of which 49 were carried out by an NS team and 70 by an NNS team. In total, the NS group had clinical events in 57% of transports and the NNS group in 77%. Endotracheal tube events (unintended extubation or tube blockage requiring reintubation) were documented in 30% of the NNS transfers and none of the NS transfers. There was no statically significant difference between the groups for hypo- or hyperthermia, hypoglycemia, or treated hypotension (the need for a fluid bolus or inotropes). A significant limitation of this study is the lack of long-term outcome data or short-term indicators of transport quality, such as arrival vital signs and blood gas parameters.

Although the numbers are small, Gardiner et al demonstrate that NS teams have fewer adverse clinical events associated with their transfers and in particular fewer airway-related events. Managing a neonatal airway is complex and distinct from pediatric and adult airway-management skills, and it is therefore reassuring that teams that included specialist neonatal nurses had fewer incidents. Of note, the NS group had significantly more ventilated babies, and it is not clear whether this is attributable to patient need or whether the teams with enhanced airway skills chose to electively intubate infants who might otherwise have been transferred with noninvasive ventilation. This study was not set up to assess the impact on parents; however, it is reasonable to assume that a reduction in adverse incidents would have a positive effect on parental experience and reduce cognitive load for the team to enable better pastoral support for the parents.

In this study, both types of teams operated within the structure of a regional neonatal transport service, benefited from a management discussion via conference call, ongoing remote support from a neonatal transport consultant (physician), and made use of standardized specialist transport equipment. In other contexts where no such service exists, these would come as a package together with the clinical expertise of the transport team members, and this study does not provide evidence for the potential benefits to patients from this complete package.

Retrospective studies are limited by the accuracy of the data recorded, potential for selection bias, and completeness of recorded data. However, ethical and practical considerations make it unlikely that we will ever have prospective randomized controlled trials demonstrating the superiority of NS transport teams. The evidence we do have is therefore likely to always be opportunistic and pragmatic, and this study makes good use of a rare opportunity to add to the evidence base. Together with findings from pediatric intensive care teams showing decreased mortality when specialist teams are used,^10^ in most contexts this lends significant weight to the argument for commissioning NS transport services. Secondarily, where considerations such as economics, geography, or case numbers preclude the introduction of a NS service, this study highlights airway management during transport as a potential target for an intervention such as high-definition simulation training to improve the performance of nonspecialist teams.

## CRediT authorship contribution statement

**Lee Collier:** Conceptualization, Investigation, Methodology, Supervision, Writing – original draft, Writing – review & editing. **Burak Salgin:** Conceptualization, Investigation, Methodology, Supervision, Writing – review & editing.

## Declaration of Competing Interest

L.C. is neonatal clinical lead for Capital Air Ambulance, a private provider of specialist aeromedical transport in the United Kingdom; Medical Director of Lia’s Wings children’s air ambulance charity; and technical advisor to Advanced Healthcare Technology Ltd., a manufacturer of neonatal transport equipment. B.S. reports no conflicts of interest.
